# Use and caregiver-reported efficacy of medical cannabis in children and adolescents in Switzerland

**DOI:** 10.1007/s00431-021-04202-z

**Published:** 2021-07-26

**Authors:** Kathrin Zürcher, Carole Dupont, Peter Weber, Sebastian Grunt, Ilca Wilhelm, Daniela E. Eigenmann, Martina L. Reichmuth, Manfred Fankhauser, Matthias Egger, Lukas Fenner

**Affiliations:** 1 Institute of Social and Preventive Medicine, University of Bern, Mittelstrasse 43, 3012 Bern, Switzerland; 2 Department of Neuropediatric and Developmental Medicine, University Children’s Hospital Basel, University of Basel, Basel, Switzerland; 3 Department of Neuropediatric, Development, and Rehabilitation, University Children’s Hospital, Inselspital, Bern University Hospital, University of Bern, Bern, Switzerland; 4 Department of Anaesthesiology and Pain Medicine, Inselspital, Bern University Hospital, University of Bern, Bern, Switzerland; 5 Bahnhof Apotheke Langnau AG, Langnau, Switzerland; 6 Population Health Sciences, Bristol Medical School, University of Bristol, Bristol, UK

**Keywords:** Medical cannabis, Cannabidiol, Children, Tetrahydrocannabinol, Chronic conditions, Treatment, Seizures

## Abstract

Evidence on the use and efficacy of medical cannabis for children is limited. We examined clinical and epidemiological characteristics of medical cannabis treatment and caregiver-reported effects in children and adolescents in Switzerland. We collected clinical data from children and adolescents (< 18 years) who received Δ9-tetrahydrocannabinol (THC), cannabidiol (CBD), or a combination of the two between 2008 and 2019 in Switzerland. Out of 205 contacted families, 90 agreed to participate. The median age at the first prescription was 11.5 years (interquartile range (IQR) 6–16), and 32 patients were female (36%). Fifty-one (57%) patients received CBD only and 39 (43%) THC. Patients were more likely to receive THC therapy if one of the following symptoms or signs were present: spasticity, pain, lack of weight gain, vomiting, or nausea, whereas seizures were the dominant indication for CBD therapy. Improvements were reported in 59 (66%) study participants. The largest treatment effects were reported for pain, spasticity, and frequency of seizures in participants treated with THC, and for those treated with pure CBD, the frequency of seizures. However, 43% of caregivers reported treatment interruptions, mainly because of lack of improvement (56%), side effects (46%), the need for a gastric tube (44%), and cost considerations (23%).

*Conclusions*: The effects of medical cannabis in children and adolescents with chronic conditions are unknown except for rare seizure disorders, but the caregiver-reported data analysed here may justify trials of medical cannabis with standardized concentrations of THC or CBD to assess its efficacy in the young.

**Table:** 

**What is Known:** *• The use of medical cannabis (THC and CBD) to treat a variety of diseases among children and adolescents is increasing.* *• In contrast to adults, there is no evidence to support the use of medical cannabis to treat chronic pain and spasticity in children, but substantial evidence to support the use of CBD in children with rare seizure disorders.*	
**What is New:** *• This study provides important insights into prescription practices, dosages, and treatment outcomes in children and adolescents using medical cannabis data from a real-life setting.* *• The effects of medical cannabis in children and adolescents with chronic conditions shown in our study support trials of medical cannabis for chronic conditions.*

**Supplementary information:**

The online version contains supplementary material available at 10.1007/s00431-021-04202-z.

## Background

*Cannabis sativa*, commonly known as cannabis, contains more than 600 ingredients. Among them are more than 100 phytocannabinoids, which include the substance Δ9-tetrahydrocannabinol (THC) [1]. THC is best known for its psychotropic effect, but THC-containing products are also used to alleviate pain, spasticity, vomiting, nausea, and loss of appetite. Drugs containing THC have been used to treat these symptoms in children with cancer, neurodegenerative diseases, cerebral palsy, traumatic brain injury, posttraumatic stress disorders, and Tourette syndrome [2–5]. In addition to THC, cannabis contains the phytocannabinoid cannabidiol (CBD), which has anti-inflammatory, antiepileptic, antipsychotic, and anxiety-relieving properties [6]. In children, CBD has been mainly used to treat epilepsy [2, 7–10], anxiety [11, 12], and autism [12, 13]. There is substantial evidence to support the use of CBD in children with rare seizure disorders, but the evidence is lacking for other types of seizures and medical conditions [2, 7–10]. However, knowledge about the use and efficacy of medical cannabis is limited beyond these conditions. Therefore, this study provides important insights into prescription practices, dosages, and treatment outcomes in children and adolescents using medical cannabis data from a real-life setting in Switzerland.

Synthetic or natural cannabis-containing preparations with more than 1% THC are regulated as narcotic drugs in Switzerland [14]. In 2011, a revision of the Swiss Law on Narcotics and Psychotropic Substances (Narcotics Law) authorized the Federal Office of Public Health to issue exceptional licenses for the medical use of substances containing more than 1% THC [15]. In contrast, pure CBD-containing preparations do not require exceptional authorizations for the prescription to patients [15, 16]. At present, several oral THC-containing preparations are prescribed as extemporaneous formulations in Switzerland with exceptional permission from the federal authorities. Until the end of April 2019, only two pharmacies (‘Bahnhof Apotheke Langnau AG’ and ‘Apotheke zur Eiche AG’) were authorized to produce THC-containing preparations for medical use.

In a previous study, we examined the requests for medical use of cannabinoids submitted to the Federal Office of Public Health in 2013 and 2014. We found that exceptional licenses for medical use of cannabinoids increased (from 542 patients treated in 2013 to 825 in 2014), with 1193 unique patients receiving treatment with cannabinoids [17]. Only 14 patients (1.2%) were younger than 20 years. Since then, the number of children and adolescents receiving medical cannabis has increased. Here, we examined the clinical and epidemiological characteristics of medical cannabis treatment and caregiver-reported effects in children and adolescents in Switzerland from February 2008 to June 2019.

## Methods

### Study design and data collection

This is a retrospective observational study that collected data from patients’ families. We obtained a list of all children and adolescents under 18 years of age who received medical THC- or CBD-containing preparations between February 2008 and June 2019 from the ‘Bahnhof Apotheke Langnau AG’ pharmacy in Langnau, Switzerland. The children and adolescents who received medical cannabis preparations from the pharmacy in Langnau came from all regions in Switzerland. The pharmacy in Langnau was one of two pharmacies in Switzerland authorized by the authorities to provide medical cannabis, as prescribed by the treating physician. The patient list included details on the date of birth, sex, medical diagnosis, date of prescriptions, the preparations including the concentration of CBD or THC, and the patients’ and the prescribing physicians’ contact details.

We developed and piloted a standardized questionnaire in collaboration with an advisory panel consisting of two pharmacists and three clinicians with experience with medical cannabis. We piloted the German and French version of the questionnaire with caregivers for completeness and understandability. The questionnaire had three sections: (i) basic information such as sex, age, diagnosis, medications other than medical cannabis, and non-pharmacological therapies; (ii) details on the medical cannabis therapy, including the symptoms triggering therapy, type of medical cannabis, initial and current or last dosage, side effects, treatment interruptions, and treatment effects; and (iii) costs of cannabis therapy, including coverage of the expenses by health insurance or out of pocket. An English translation of the questionnaire is reproduced in the supplemental information (Table S1). We assessed treatment effects using a Likert scale with options ranging from ‘much less’, ‘less’, ‘no change’, ‘more’, to ‘much more’. The survey was provided in paper form or electronically in a REDCap application [18].

We sent all children and adolescents’ caregivers an invitation letter with information on the study, the informed consent form, and the questionnaire with a prepaid return envelope. We sent non-responders another questionnaire 4–6 weeks later. In the event of continued nonresponse, we contacted families by phone. Depending on the caregivers’ preference, either a caregiver or the adolescent (≥ 14 years old) or both filled in the questionnaire.

### Definitions

We assigned children and adolescents to two groups: those treated with a pure CBD preparation and those treated with a THC-containing preparation (with or without low concentrations of CBD). Patients treated with both THC and pure CBD were analysed in the THC group. In other words, patients who received THC, and an additional preparation of pure CBD, were included in the THC group. The medical cannabis preparations were standardized for THC or CBD concentration. The pure THC solution (dronabinol solution 2.5%) contained 0.7 mg THC per drop, the standardized alcohol-based cannabis tincture (10 mg THC/ml and 20 mg CBD/ml) 0.3 mg THC and 0.6 mg CBD per drop, and the standardized cannabis oil (10 mg THC/ml and 20 mg CBD/ml) 0.4 mg THC and 0.8 mg CBD per drop. The pure CBD solutions with concentrations of 2.5%, 5%, and 10% contained 0.7 mg, 1.4 mg, and 2.8 mg per drop, respectively [19, 20].

We categorized the diagnosis by the International Classification of Diseases (ICD-10), version 2019, into nine groups (ICD-10 codes in parentheses):

• Infectious and parasitic diseases (A00-B99).
• Cancer (C00–C97).
• Endocrine, nutritional, and metabolic diseases (E00-E90).
• Mental and behavioural disorders (F00–F99).
• Diseases of the nervous system (G00–G99).
• Diseases of the skin and subcutaneous tissue (L00-L99).
• Diseases of the musculoskeletal system and connective tissue (M00-M99).
• Congenital malformations, deformations, and chromosomal abnormalities (Q00-Q99).
• Injury, poisoning, and other conditions with external causes (S00–S99).

### Statistical analysis

We used descriptive statistics to characterize patients and treatment effects and assessed differences between groups using chi‐square, Fisher’s exact, or Wilcoxon rank‐sum tests. We assessed changes in the dosage of products using the paired t-test. All analyses were done in Stata (version 15.1, College Station, TX, USA).

### Ethics

The Cantonal Ethics Committee Bern, Switzerland, approved this study (No. 2019–00,049). Written informed consent was obtained from the caregivers of each child or adolescent younger than 14 years. Among older adolescents, either the adolescent or the caregivers gave informed consent.

## Results

**Fig. 1:**
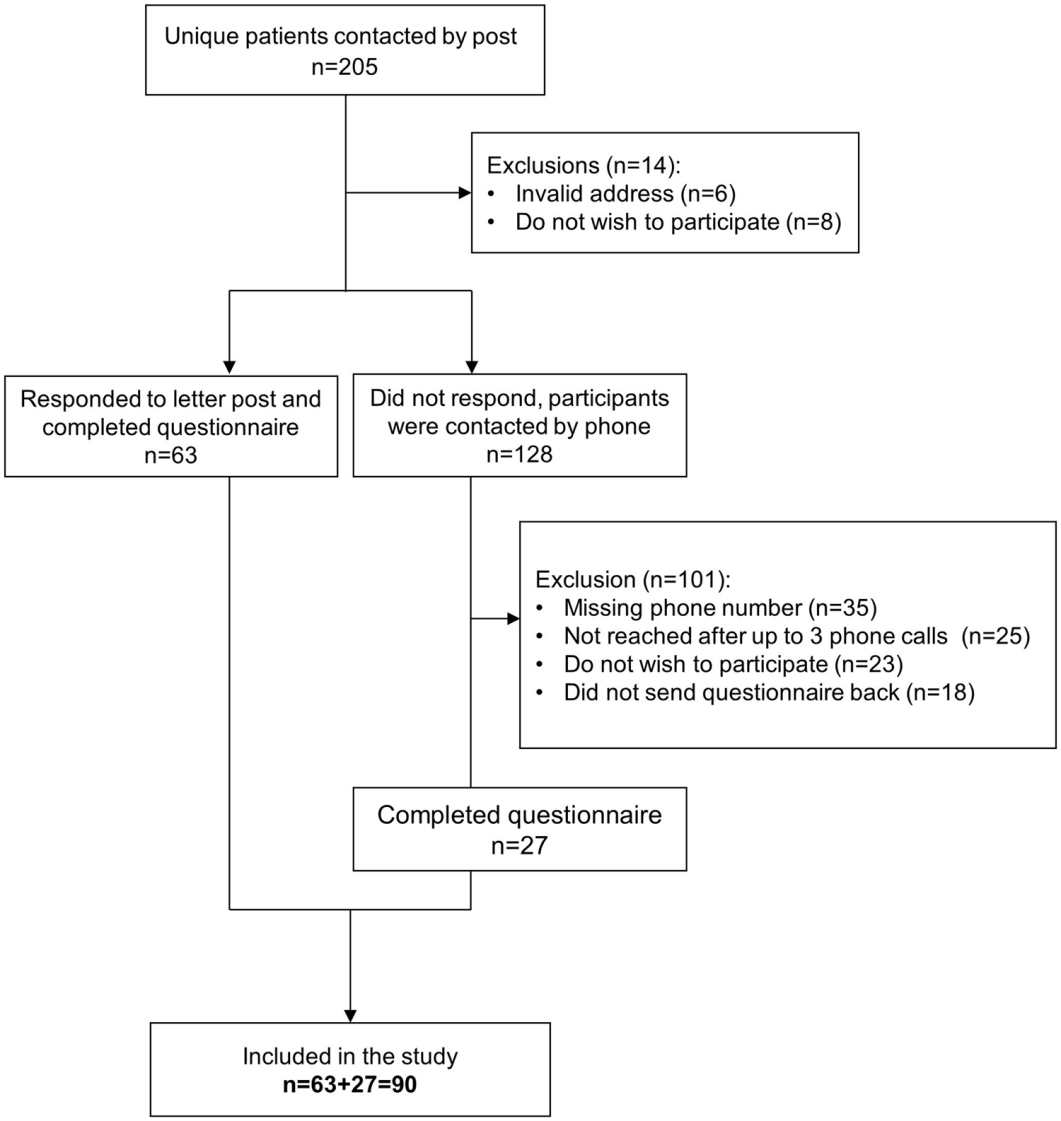
Flow of inclusion of study participants

The patient list of the pharmacy included data on 205 children or adolescents who were treated with THC- or CBD-containing preparations between February 2008 and June 2019. The first prescription to a child or adolescent was in 2013. Initially, we received 77 responses from caregivers. After the reminders, 90 caregivers (43.9%) agreed to participate in the study. Figure 1 shows the recruitment into the study. We compared the characteristics of the 90 participating children or adolescents to the 115 patients who did not participate. There were no differences in age and the type of medical cannabis prescribed. Compared to non-responders, participants were more likely to be male, to have more than one ICD-10 diagnosis, to have a disease of the nervous system or an endocrine, nutritional and metabolic disease, and have multiple prescriptions of medical cannabis products. According to the ICD-10 classification, the most common diseases among the 205 children or adolescents were diseases of the nervous system (120; 59%), mental and behavioural disorders (26; 13%), cancer (15; 7%), congenital malformations, deformations and chromosomal abnormalities (15; 7%), and endocrine, nutritional, and metabolic diseases (11; 5%). For 34 (17%), the diagnosis was missing (Table S2).

### Patient characteristics

The median age at the first prescription of the 90 participants was 11.5 years (interquartile range 6–15), and 32 were female (36%, Table 1). The youngest participant was 4 months old with a neurodegenerative disease and the oldest 17 years old with epilepsy. Both received CBD only to treat seizures. More than half of the participants (57%) suffered from more than one disease. The most common diagnosis were epilepsy (66; 73%), cerebral palsy (32; 36%), encephalopathy (15; 17%), metabolic diseases (8; 9%), and autism (7; 7%). Among the 66 participants with epilepsy, 24 (36%) had only epilepsy, whereas 42 (64%) had epilepsy with additional diseases (Table S3).

**Table 1:** Characteristics of the 90 included participants

	Total	THC^a^	CBD only	*p* value
Total	*n* = 90 (100%)	*n* = 39 (100%)	*n* = 51 (100%)	
**Sex**				0.62
Male	58 (64)	24 (62)	34 (67)	
Female	32 (36)	15 (38)	17 (33)	
**Median age at the first prescription in years (IQR)**	11.5 (6–15)	14 (9–16)	9 (6–14)	0.029
**Number of diagnoses**				0.09
One	39 (43)	13 (33)	26 (51)	
Two or more	51 (57)	26 (67)	25 (49)	
**Diagnosis**^b^				
Epilepsy	66 (73)	22 (56)	44 (86)	0.001
* Drug-resistant epilepsy *	*20 (22)*	*3 (8)*	*17 (33)*	
* Dravet syndrome *	*7 (8)*	*1 (3)*	*6 (12)*	
* Lennox–Gastaut syndrome (LGS)*	*7 (8)*	*-*	*7 (14)*	
* Absences*	*4 (4)*	*3 (8)*	*1 (2)*	
Cerebral palsy	32 (36)	19 (49)	13 (26)	0.071
Encephalopathy	15 (17)	6 (15)	9 (18)	0.78
Metabolic disease	8 (9)	6 (15)	2 (4)	0.07
Autism	7 (8)	1 (3)	6 (12)	0.13
Genetic disorder	6 (7)	4 (10)	2 (4)	0.40
Cancer	4 (4)	4 (10)	0	0.03
Tourette’s syndrome	3 (3)	2 (5)	1 (2)	0.58
Severe head injury	3 (3)	3 (8)	0	-
Other^c^	8 (9)	4 (10)	4 (8)	
**Symptoms/indication**^b^				
Seizure/epilepsy	60 (67)	20 (51)	40 (78)	0.007
Spasticity	27 (30)	20 (51)	7 (14)	0.001
Pain	26 (29)	20 (51)	6 (12)	0.001
Sleep disorder	15 (17)	5 (13)	10 (20)	0.39
Lack of weight gain	11 (12)	8 (21)	3 (6)	0.05
Anxiety disorders/behaviour	10 (11)	7 (18)	3 (6)	0.10
Vomiting	9 (10)	7 (18)	2 (4)	0.04
Nausea	8 (9)	7 (18)	1 (2)	0.02
Loss of appetite	7 (8)	6 (15)	1 (2)	0.04
ADHD, behaviour change	5 (6)	1 (3)	4 (8)	0.38
Inflammatory condition	4 (4)	2 (5)	2 (4)	1.0
Tics	3 (3)	2 (5)	1 (2)	0.58
Others	3 (3)	3 (8)	-	-
**Type of medical cannabis preparation, initial**				-
THC-based preparation	39 (43)	39 (100)		
* Dronabinol solution 2.5%*	*20 (22)*	*20 (51)*	*-*	
* Standardized cannabis tincture*	*10 (11)*	*10 (26)*	*-*	
* Standardized cannabis oil*	*9 (10)*	*9 (23)*	*-*	
CBD-based preparation	51 (57)		51 (100)	
* CBD 2.5%*	*20 (22)*	*-*	*20 (39)*	
* CBD 5%*	*17 (19)*	*-*	*17 (33)*	
* CBD 10%*	*14 (16)*	*-*	*14 (28)*	
**Other co-medications**^b^				
Antiepileptic drugs	60 (67)	21 (5)	39 (77)	0.024
Muscle relaxants	10 (11)	8 (21)	2 4)	0.018
Analgesics and opiates	10 (11)	9 (23)	1 (2)	0.002
Other drugs	25 (28)	15 (39)	11 (22)	0.08
**Additional therapy**^b^				
Physical therapy	63 (70)	30 (77)	33 (65)	0.21
Occupational therapy	46 (51)	17 (44)	29 (57)	0.21
Osteopathy	23 (26)	10 (26)	13 (26)	0.99
Speech therapy	9 (10)	4 (10)	5 (10)	0.94
Traditional Chinese Medicine (TCM)	6 (7)	2 (5)	4 (8)	0.61
Homeopathy	5 (6)	2 (5)	3 (6)	0.88
Chiropractic	5 (6)	2 (5)	3 (6)	0.88
Others	11 (12)	5 (13)	6 (12)	0.88
No answer	13 (14)	5 (13)	8 (16)	0.90

*CBD* cannabidiol, *THC* tetrahydrocannabinol, *ADHD* attention-deficit/hyperactivity disorder

^a^Six patients who received both THC and pure CBD were assigned to THC

^b^*p* values for each diagnosis vs. no disease (reference), each symptom vs. no symptom (reference), and each additional therapy vs. no additional therapy (reference), each co-medication vs. no other co-medication were calculated using chi-squared of Fisher exact

^c^Other diseases include 2 with neuropathic pain, 2 with AD(H)S, 2 with epidermolysis sclerosis, 1 with depression, 1 with tuberous sclerosis

**Fig. 2:**
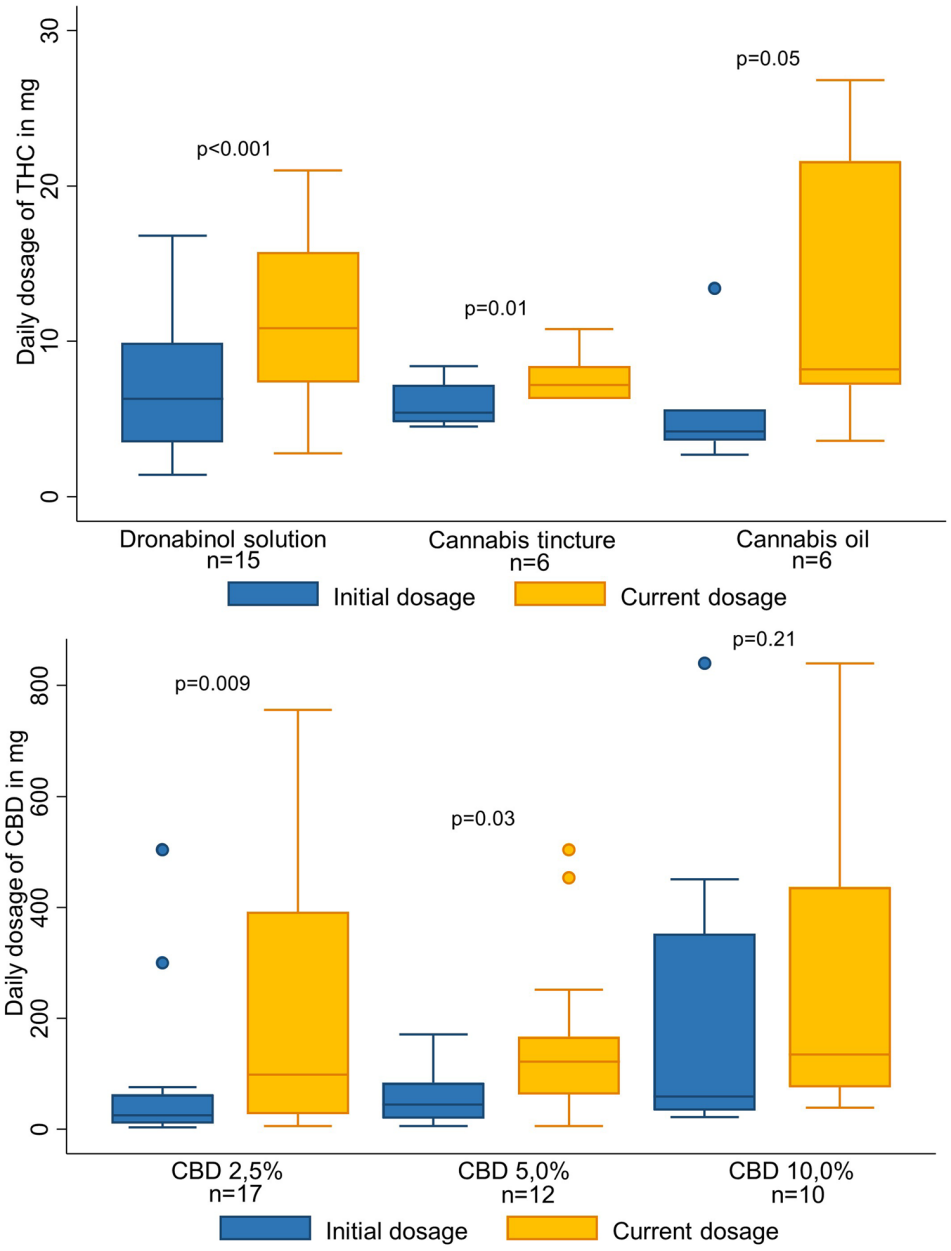
Changes of the prescribed medical cannabis preparation from initial to current dosage by patients for whom initial and current dosage was available

Fifty-one participants (57%) were treated with CBD only and 39 (43%) with a THC preparation. Six patients who received a THC-containing preparation and pure CBD (three received dronabinol and 2.5%, 5%, or 10% pure CBD; two cannabis tincture and 5% or 10% pure CBD; and one cannabis oil and 2.5% CBD) were included in the THC group. When analysing the groups ‘THC only’ and ‘THC and CBD’, we found no statistical difference (Table S4). THC was more commonly prescribed to participants with cancer (*p* = 0.03), whereas CBD only was more frequently prescribed to participants with epilepsy (*p* < 0.001). Participants were more likely to receive THC therapy if one of the following symptoms or signs were present: spasticity, pain, lack of weight gain, loss of appetite, vomiting, or nausea, whereas seizures were the dominant indication for CBD only therapy. The daily dosage of medical cannabis preparations increased over time for both THC- and CBD-only preparations (Fig. 2).

The majority of participants (72; 80%) received at least one concomitant medication. The most frequent medication categories were antiepileptic drugs (60; 67%), followed by muscle relaxants (10; 11%), analgesics (10; 11%), and other drugs (25; 28%). Also, 63 (70%) participants received physical therapy, 46 (51%) occupational therapy, and 23 (26%) osteopathy (Table 1).

### Treatment interruption and side effects

During medical cannabis treatment, 39 of the 90 participants (43%) reported a treatment interruption (Tables 2 and S5). Twenty-two stopped treatment definitively, and 17 resumed treatment (six continued with the same preparation and dosage, seven continued with the same preparation but a different dosage, four continued with another preparation). The median time from treatment initiation to treatment interruption was eight weeks (IQR 3–32 weeks). The reasons given for the treatment interruption included lack of improvement in 22 patients (56%), side effects in 18 (46%), the need for a gastric tube in 17 cases (44%) preventing the continuation of treatment, and cost considerations in 9 patients (23%, Tables 2 and S5). Side effects were observed in 25 of the 90 participants (28%) and similar in the THC and CBD group. The three most common side effects were tiredness, sedation, and dry mouth (Tables 2 and S5).

**Table 2:** Outcomes of medical cannabis therapy among 90 children and adolescents

	Total *n* = 90 (100%)		THC^a^ *n* = 39 (100%)	CBD only *n* = 51 (100%)	*p* value
**Treatment success (caregiver perspective)**					0.65
Yes	59 (66)		26 (67)	33 (65)	
No	28 (31)		11 (28)	17 (33)	
Missing	3 (3)		2 (5)	1 (2)	
**Treatment interruptions/treatment stop**					0.70
Yes	39 (43)		16 (41)	23 (45)	
No	51 (57)		23 (59)	28 (55)	
**Median time to first interruption/treatment stop in weeks (IQR)**	8 (3–32)		6.5 (3.5–36)	8 (3–20)	
* Number of observations*	*25*		*8*	*17*	
**Reasons for interruption**^b^					
No improvement and stopped treatment	22 (24)		9 (23)	13 (26)	0.79
Side effects	18 (20)		8 (21)	10 (20)	0.92
Taking preparation via the tube	17 (19)		7 (18)	10 (20)	0.84
Costs	9 (10)		0	9 (18)	0.004
Unpleasant smell/taste	4 (4)		2 (5)	2 (4)	1
**Side effects**					0.94
No	65 (72)		28 (72)	37 (73)	
Yes	25 (28)		11 (28)	14 (27)	
* Tiredness*	*11 (12)*		*6 (15)*	*5 (10)*	
* Sedation*	*7 (8)*		*4 (10)*	*3 (6)*	
* Dry mouth*	*5 (6)*		*2 (5)*	*3 (6)*	
* Nausea and vomiting*	*4 (4)*		*2 (5)*	*2 (4)*	
* Dizziness*	*2 (2)*		*2 (5)*	*0*	
* Hallucinations*	*2 (2)*		*1 (3)*	*1 (2)*	
* Impaired ability to think (cognitive changes)*	*2 (2)*		*2 (5)*	*0*	
* Changed movement behaviour*	*2 (2)*		*1 (3)*	*1 (2)*	
* Diarrhoea*	*2 (2)*		*0*	*2 (4)*	
* Red eyes*	*1 (1)*		*1 (3)*	*0*	
* Others*	*6 (7)*		*0*	*6 (12)*	

*CBD* cannabidiol, *THC* tetrahydrocannabinol

^a^Six patients who received both THC and pure CBD were assigned to THC

^b^*p* values for each reason for interruption vs. no interruption (reference)

### Awareness, prescription, and cost modalities

Caregivers learned about medical cannabis therapy through the media (44; 49%), their family doctor or medical specialist (37; 41%), and friends or family members (21; 23%). In most cases (82; 91%), specialist doctors, neuropaediatricians, neurologists, oncologists, or palliative care specialists prescribed the preparation. The cost of the first prescription was reimbursed by the invalidity insurance (insurance covering some chronic diseases such as epilepsy or cerebral palsy) in 50 participants (56%), and the health insurance covered the cost for ten patients (11%, Table 3). For 27 participants (30%), caregivers paid out of their pocket. This situation persisted during the treatment. The monthly cost was below 300 USD for 24 participants (27%), between 301 to 600 USD for 22 (24%), more than 600 USD for 25 (28%). The cost was unknown for the remaining 19 children or adolescents. Many caregivers were concerned about the high cost of medical cannabis preparations. Table S3 gives further details about costs.

**Table 3:** Sources of coverage of medical cannabis in USD

	Total *n* = 90 (100%)	THC^a^ *n* = 39 (100%)	CBD only *n* = 51 (100%)	*p* value
**Initial cost coverage**				0.17
Invalidity Insurance	50 (56)	22 (56)	28 (55)	
Out of pocket	27 (30)	8 (21)	19 (37)	
Health insurance	10 (11)	8 (21)	2 (4)	
Other (support-group)	1 (1)	0	1 (2)	
Unknown	2 (2)	1 (3)	1 (2)	
**Current cost coverage**				
Invalidity Insurance	54 (60)	24 (62)	30 (59)	0.26
Out of pocket	24 (27)	7 (20)	17 (33)	
Health insurance	9 (10)	7 (20)	2 (4)	
Other (support-group)	1 (1)	0	1 (2)	
Unknown	2 (2)	1 (3)	1 (2)	
**Exact monthly amount, median (IQR)**	360 (212–1250)	250 (140–1250)	500 (250–1400)	0.49
* Number of observations*	33	11	22	
**Monthly costs**				0.051
< 100 USD	4 (4)	3 (8)	1 (2)	
100–300 USD	20 (22)	8 (21)	12 (26)	
301–600 USD	22 (24)	8 (21)	14 (27)	
> 600 USD	25 (28)	7 (18)	18 (35)	
Unknown	19 (21)	13 (33)	6 (12)	

*CBD* cannabidiol, *THC* tetrahydrocannabinol

^a^Six patients who received both THC and pure CBD were assigned to THC

### Treatment effects of medical cannabis preparations

**Fig. 3:**
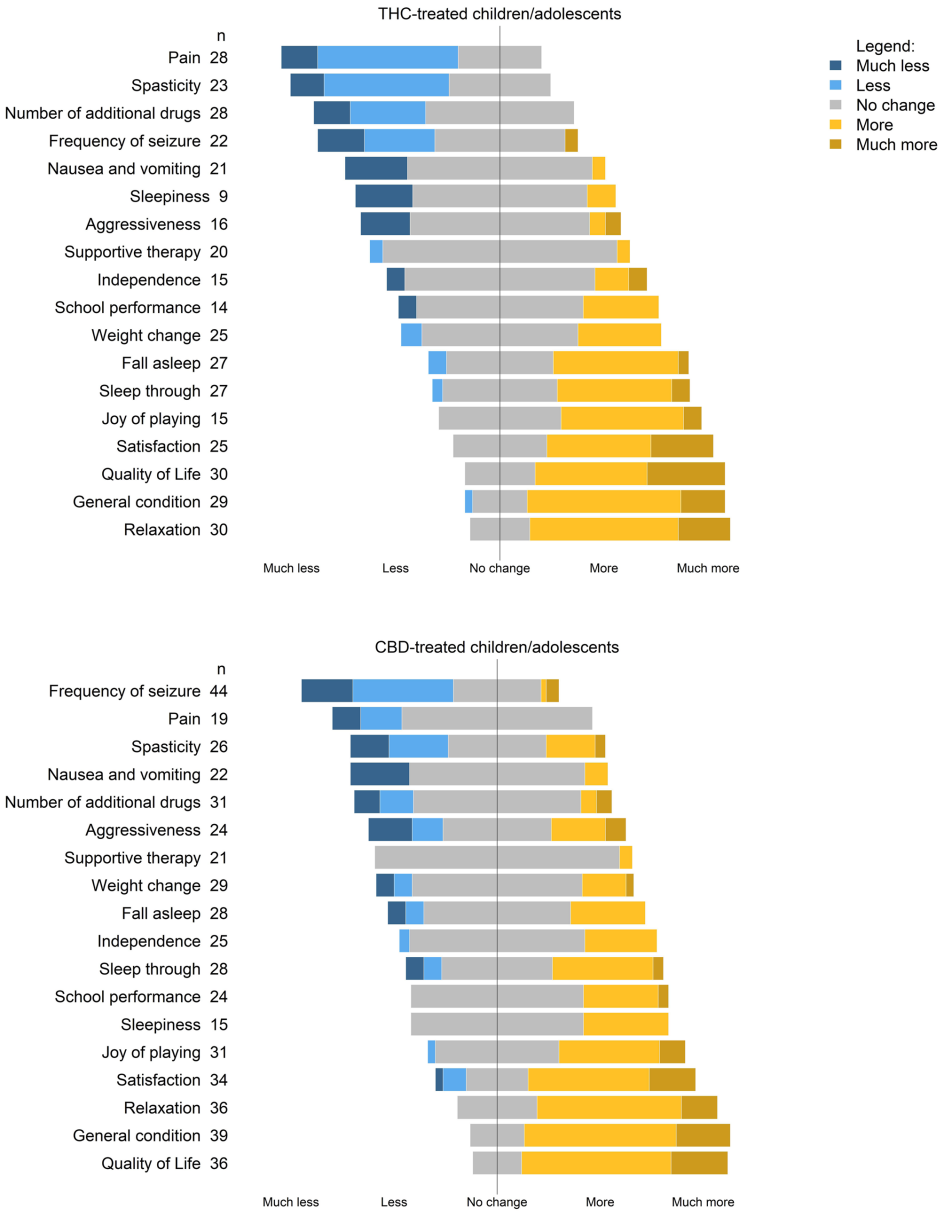
Reported treatment effects of medical cannabis use in children and adolescents from the caregivers’ perspective. The number indicates the number of responses. Detailed results are presented in additional Table 3

In 59 of 90 participants (66%), the treatment with medical cannabis was reported to be successful by the caregivers (Tables 2 and S5). Participants treated with THC products most frequently reported a reduction of pain, spasticity, seizures, and a reduction in the number of drugs taken. Participants treated with pure CBD-containing products most commonly reported a reduction in the frequency of seizures. Irrespective of treatment with THC or CBD only, caregivers felt that their children or adolescents were more relaxed, more satisfied, and in better general condition than before the therapy with medical cannabis (Fig. 3, Table S6). The use of other therapies like physiotherapy, osteopathy, speech, or occupational therapy did not change, regardless of whether the patients received THC or CBD only (Fig. 3).

## Discussion

The use of medical cannabis to treat a variety of diseases among children and adolescents is increasing. We found that THC was most frequently used to treat pain, spasticity, seizures, lack of weight gain, and nausea, while CBD only was used to treat pain, seizures, and sleep disorders. The largest treatment effects were reported for pain, spasticity, and frequency of seizure in participants treated with THC, and for those treated with CBD only, the frequency of seizures. Irrespective of treatment with THC or CBD only, or a preparation containing both, most caregivers felt that their children and adolescents were more relaxed, more satisfied, and had increased quality of life with medical cannabis treatment. The treatment was reported to be successful in two thirds of participants. Treatment interruption was frequent, mainly due to lack of improvement, side effects, or cost considerations.

In our study, 80% of participants suffered from a disease of the nervous system. The most common diagnosis was epilepsy, for which 55% of caregivers reported a reduction in seizures and 6% reported seizure aggravation while on medical cannabis. In a similar study examining treatment with cannabidiol-enriched cannabis in children with treatment-resistant epilepsy, 84% of caregivers reported a reduction in the frequency of their children’s seizures [21]. Another retrospective study based on clinical records showed that children with intractable epilepsy treated with CBD-enriched cannabis oil with a 20:1 CBD to THC formulation reported a reduction in the frequency of seizure in almost 90% of children, along with aggravation of seizures in 7% [22]. Beyond observational studies, randomized controlled trials (RCT) were conducted in children with treatment-resistant epilepsy to assess the efficacy and tolerability of CBD compared to placebo. These studies confirmed that CBD reduces seizure frequency [7–10]. A study from Israel showed that epilepsy in children can also be treated with a standardized preparation containing CBD and THC [23]. Finally, a recent systematic review and meta-analysis summarized results from intervention studies and concluded that CBD is more effective than placebo for treatment-resistant epilepsy, regardless of the aetiology of the epileptic syndrome [24].

The second most common indication for the use of medical cannabis was spasticity. Among our participants, 49%, mainly those with cerebral palsy, reported a reduction of spasticity. A case series of 16 children, adolescents or young adults with resistant spasticity in palliative care who received 2.5% dronabinol showed reduced spasticity [25]. An intervention study in children with complex motor disorders treated either with cannabis oil with a 20:1 or 6:1 CBD to THC formulation showed similar improvements in both groups in spasticity, sleep difficulties, pain severity, and quality of life [26]. However, a recent multicentre RCT (2020) in 72 children or adolescents with cerebral palsy or another central nervous system injury after birth found no significant difference in spasticity with a cannabis extract (Nabiximols) and placebo after 12 weeks of treatment (3). Today, the evidence that medical cannabis has an impact on spasticity in children is weak. In adults with multiple sclerosis or paraplegia, a systematic review of RCTs showed some evidence supporting the efficacy of medical cannabis in spasticity [27–30].

he use of medical cannabis in children and adolescents poses risks. Our study found that medical cannabis was prescribed for various conditions, even though the evidence is weak for many. Although there is some evidence in adults supporting the efficacy of medical cannabis for some diseases, we should not extrapolate results from adults to children. Limited evidence exists for the effective use of different cannabis derivatives, dosage, and indications. Indeed, the high rate of treatment interruption or stop (43%) seen in our study was driven by side effects and lack of improvement. Thus, clinicians should closely monitor children and adolescents on medical cannabis for efficacy and adverse effects, and they should be experienced in the treatment of the underlying disease. In addition, potential drug interactions should be considered. A systematic review on safety and efficacy in epilepsy summarized drug–drug interactions of CBD with other drugs metabolized in the liver by cytochrome P450 enzymes exists. Therefore, treatment with CBD can result in elevated liver enzyme, especially when co-medicated with the antiepileptic drug valproate [31]. However, current evidence is limited about both the interaction of medical cannabis with other drugs. Treatment guidelines are needed to inform decision making by clinicians and caregivers. Although such guidelines are available in a few countries [32–34], they currently do not exist in Switzerland and many other countries. Another reason for stopping treatment was the costs for medical cannabis, which have to be paid out of the caregivers’ pocket. The production of quality-controlled medical cannabis is laborious. The costs for other medical and nonmedical treatments in these children, which will generally be covered by health insurance, might be substantially higher [35].

This is one of the first studies reporting on prescription practices, dosages, and treatment outcomes in a large sample of children and adolescents using medical cannabis in a real-life setting. Two strengths particularly distinguish our study from others. Participants were patients under 18 years of age who received medical cannabis over the entire period since its use was authorised in Switzerland. The pharmacy in Langnau is a pioneer in this field and the largest professional distributor of medical cannabis in Switzerland. Also, even though the dosage of medical cannabis preparations varied, the concentrations were standardized. Other publications and recommendations often lack such standardization, which hampers comparisons across studies.

The most important limitation of our study was the lack of a comparison group. Another limitation derives from the fact that the caregivers reported the data, which may lead to a recall bias. However, it is common in the paediatric field to collect outcome data reported by caregivers, especially in children with disabilities. A further limitation is the potential selection bias arising from group differences between those who participated and those who did not. Participants’ health and the situations of participating families may have been better than those of nonparticipants. How this could have influenced our findings is unknown.

## Conclusions

In Switzerland, medical cannabis was mainly prescribed to children and adolescents with neurological diagnoses, particularly epilepsy. Although evidence supporting efficacy is lacking, medical cannabis was prescribed to children and adolescents for various other conditions. For two thirds of participants treated with standardized THC or CBD preparations, the caregiver reported an improvement in their condition and well-being. Others stopped preparations because of lack of effectiveness or side effects. Medical cannabis could be a promising and useful therapy for children and adolescents with neurological conditions. However, we need further RCTs with standardized THC and CBD preparations to assess the efficacy of medical cannabis in different diseases and long-term effects in the young.

## Supplementary information

Below is the link to the electronic supplementary material.

Supplementary file1 (DOCX 58 KB)

## Data Availability

The collected data and the datasets used for this study are available from the corresponding author on reasonable request.

## Abbreviations

CBD: Cannabidiol
CI: Confidence interval
IQR: Interquartile range
RCT: Randomized controlled trials
THC: Δ9-Tetrahydrocannabinol
