# Supplementary material for: Use and caregiver-reported efficacy of medical cannabis in children and adolescents in Switzerland
Source: Eur J Pediatr. 2021 Jul 26;181(1):335–47. doi: 10.1007/s00431-021-04202-z (PMC8760226; doi:10.1007/s00431-021-04202-z)
Supplement: Supplementary file 1 — Supplementary file1 (DOCX 58 KB) [file 431_2021_4202_MOESM1_ESM.docx]

**Supplementary Information**

**Table S1:** Questionnaire


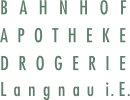

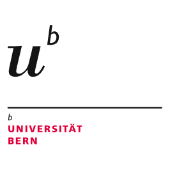
QUESTIONNAIRE FOR THE CAREGIVERS OF THE AFFECTED

CHILDREN / ADOLESCENTS

Study participant ID: ……………………………………………………..

1. **BASIC DATA**

1. What diagnosis did the doctors find in your child? (Several answers possible)

| - Spastic cerebral palsy - Dystonic cerebral palsy - Hemi-, para-, tetra-, or quadriplegia^^[[1]](#footnote-1)^^ - Encephalopathy - Epilepsy - Tourette's syndrome - Cancer | - Autism - AD(H)S - Depression - Migraine - Tuberous sclerosis - Epidermolysis bullosa - Other: ………………………………………..……… . |
| --- | --- |

1.1 If cancer: What type of cancer does/did your child suffer from?

| - Leukemia - Brain and/or spinal cord cancer - Hodgkin's lymphoma - Non-Hodgkin's lymphoma | - Soft tissue cancer - Bone cancer - Others:………………………………………………… . |
| --- | --- |

1.2 If epilepsy: Which type of epilepsy?

- Focal seizures: What sub form? ..........................................................................................
- Generalized seizures: What sub form? ……………………………………………………………………………....
- Combined focal/generalized seizures: What shape? …………………………………………………………....
- Dravet syndrome
- Lennox-Gastaut Syndrome (LGS)
- Pharmacoresistant epilepsy
- Others: …………………………………………………………………...

1.3 If epilepsy: What is the cause of epilepsy?

| - Idiopathic - Genetic | - Structural - Others: ………………………………………………… |
| --- | --- |

1.4 If epilepsy: Can you remember how often your child had epileptic seizures before starting cannabis therapy?

| - Daily - Several times a week - 1-2 times per month | - Several times a year - Others: ………………………………………………… |
| --- | --- |

2. Did the doctors find any other secondary diagnosis/comorbidities in your child?

- No
- Yes, which ones? ………………………………………………………………………………………………………………….

1. **DRUG THERAPY**

3. What drug(s) did your child receive before starting cannabis therapy? (Please indicate medicine(s) and dosage(s))

Before:………………………………………………………………………………………………………………………………….. Current: …………………………………………………………………………………………………………………………………

4. In addition to drug therapy, did your child receive any other therapies before starting cannabis therapy? (Multiple answers possible)

| - Physical therapy - Occupational therapy - Osteopathy | - Traditional Chinese Medicine (TCM) - Chiropractic - Others: ………………………………………………… |
| --- | --- |

1. **CANNABIS THERAPY**

5. How did you become aware of cannabis therapy? (Multiple answers possible)

| - Family doctor^^[[2]](#footnote-2)^^: FMH …………………………… - Specialist^^[[3]](#footnote-3)^^: FMH ……………..…………………… - From other affected persons | - Friends/colleagues - Media (Internet, newspaper, television, radio, specialist literature, etc.) - Others: ………………………………………………… |
| --- | --- |

6. What are/were the symptoms for the indication of cannabis therapy? (Multiple answers possible)

| - - Acute pain   - Chronic pain   - Spasticity   - Sickness   - Vomiting   - Anxiety states | - - Seizures   - Loss of appetite   - Lack of weight gain   - Inflammatory condition   - Other: ………………………………………………. |
| --- | --- |

7. In what form is/have the cannabis preparations been administered to your child? Please indicate dosage per day e.g. 3x10ml, 1x10ml. (Multiple answers possible)

| **Cannabis preparation** |  | **Dosage per day in drops, ml or mg** | |
| --- | --- | --- | --- |
|  |  | Initial | Current / Last |
| Dronabinol solution 2.5%. |  |  |  |
| Standardized Cannabis tincture (10 mg THC/ml, 20 mg CBD/ml) |  |  |  |
| Standardized Cannabis oil (10 mg THC/ml, 20 mg CBD/ml) |  |  |  |
| Cannabidiol (CBD) solution 2.5% |  |  |  |
| Cannabidiol (CBD) Solution 5% |  |  |  |
| Cannabidiol (CBD) Solution 10% |  |  |  |
| Sativex |  |  |  |
| Other:…………………………………………………….. |  |  |  |

8. Were any side effects observed during cannabis therapy?

- No
- Yes

8.1 If yes, what were/are the side effects? (Multiple answers possible)

| - Fatigue - Diarrhea - Vomiting - Sickness - Vertigo / Dizziness - Red eyes - Dry mouth | - Hallucinations - Drowsiness (sedation) - Changes in movement behavior - Impaired ability to think (cognitive changes) - Others: ………………………………………………… |
| --- | --- |

9. Were there any treatment interruptions or a treatment stop during cannabis therapy?

- Yes, there were therapy interruptions. Number of interruptions: ………………………………………
- No, there was no therapy interruptions.

9.1 In case of treatment interruptions or treatment stop, in which week after the start of the cannabis therapy were these?

| 1. Interruption / treatment stop:…………………………………………………………………………………………………. |
| --- |
| 2. Interruption / treatment stop:……………………………………………………………………….………………………… |
| 3. Interruption / treatment stop:……………………………………………………………………………………………….… |
| 4. or more interruption / treatment stop ……………………………………………………………………………………. |

9.2 If yes, what are/were the reasons for the treatment stop? (Multiple answers possible)

- No improvement in symptoms
- Side effects
- Therapy costs too expensive
- Odor of the cannabis preparation unpleasant
- Taking the cannabis preparation as a liquid was unpleasant (e.g. too oily)
- Taking the cannabis preparation via the gastric tube
- Others: ……………………………………………………………………………………………………………………………

9.3 After treatment interruption(s), was cannabis therapy continued?

- Yes, the cannabis therapy is/was continued with the same cannabis preparation and the same dosage.
- Yes, the cannabis therapy is/was continued with the same cannabis preparation but with a different dosage.
- Yes, the cannabis therapy is/was continued with another cannabis preparation.
- No, cannabis therapy was definitely stopped.

1. **TREATMENT RESULT**

10. Do you think that cannabis therapy has been successful in your child?

- Yes
- No
- Was successful, but was stopped. Reason: ……………………………………………………………………….

11. After starting cannabis therapy, the following changes were observed in your child *compared to the time before cannabis therapy*. (Multiple answers possible)

|  | Much more | more | Less | Much less | No change | Not applicable |
| --- | --- | --- | --- | --- | --- | --- |
| Epileptic seizure |  |  |  |  |  |  |
| Number of epileptic seizures per day/week/month before^^[[4]](#footnote-4)^^ therapy: …………. After therapy: …………… | | | | | | |
| Falling asleep |  |  |  |  |  |  |
| Sleeping through |  |  |  |  |  |  |
| Weight change |  |  |  |  |  |  |
| Number of additional drugs |  |  |  |  |  |  |
| Other therapy |  |  |  |  |  |  |
| Aches |  |  |  |  |  |  |
| Nausea and vomiting |  |  |  |  |  |  |
| Others: …………………………… |  |  |  |  |  |  |

12. After starting cannabis therapy, the following subjective changes were observed in your child *compared to the time before cannabis therapy*. (Multiple answers possible)

|  | Much more | more | Less | Much less | No change | Not applicable |
| --- | --- | --- | --- | --- | --- | --- |
| Spasticity |  |  |  |  |  |  |
| Autonomy |  |  |  |  |  |  |
| Aggressiveness |  |  |  |  |  |  |
| Relaxation |  |  |  |  |  |  |
| School achievements |  |  |  |  |  |  |
| Satisfaction |  |  |  |  |  |  |
| Joy in playing |  |  |  |  |  |  |
| General condition |  |  |  |  |  |  |
| Quality of life overall |  |  |  |  |  |  |
| Other: ………………………… |  |  |  |  |  |  |

1. **PAYMENT MODALITIES**

13. Who *initially* covered the costs for cannabis therapy?

| - Self-payer - Health insurance | - Disability insurance - Others: ……………………………………………… |
| --- | --- |

14. What are the current total monthly costs for the cannabis therapy?

⬜⬜⬜⬜ . ⬜⬜ CHF per month (average)

14.1 If you do not know the current total monthly costs, you may give us the a cost range (per

month)

| - <100 CHF - 100-300 CHF | - 301-600 CHF - >600 CHF |
| --- | --- |

16. Who covered the cost of cannabis therapy *most recently*?

| - Self-payer - Health insurance | - Disability insurance - Others: ……………………………………………… |
| --- | --- |

1. **FINAL COMMENTS**

Here you can write any additional comments.

……………………………………………………………………………………………………………………………………………………………. …………………………………………………………………………………………………………………………………………………………….

…………………………………………………………………………………………………………………………………………………………….

**Table S2:** Characteristics of the participating and not participating children and adolescents

|  | **All**  n=205 (100%) | | **Participating**  n=90 (100%) | | **Not participating**  n=115 (100) | | **p-value** | |  |
| --- | --- | --- | --- | --- | --- | --- | --- | --- | --- |
| **Sex** |  | |  | |  | | 0.004 | |  |
| Male | 109 (53) | | 58 (64) | | 51 (44) | |  | |  |
| Female | 96 (47) | | 32 (36) | | 64 (56) | |  | |  |
| **Median age** **at the first prescription in years (IQR)** | 12 (7-15) | | 12 (7-15) | | 11.5 (6-15) | | 0.59 | |  |
| **Type of medical cannabis** |  | |  | |  | |  | |  |
| THC | 84 (41) | | 33 (37) | | 51 (44) | |  | |  |
| CBD | 112 (55) | | 51 (57) | | 61 (53) | |  | |  |
| THC and pure CBD | 9 (4) | | 6 (7) | | 3 (3) | |  | |  |
| **Prescription** |  | |  | |  | | 0.02 | |  |
| Single | 35 (17) | | 9 (10) | | 26 (23) | |  | |  |
| Multiple | 170 (83) | | 81 (90) | | 89 (77) | |  | |  |
| **Number of ICD-10 diagnoses** ^1^ |  | |  | |  | | 0.007 | |  |
| One | 146 (71) | | 69 (77) | | 77 (70) | |  | |  |
| Two or more | 25 (12) | | 21 (23) | | 4 (3) | |  | |  |
| Missing | 34 (17) | | - | | 34 (30) | |  | |  |
| **Categorization of the diagnosis by ICD10**  ^2^ |  | |  | |  | |  | |  |
| Diseases of the nervous system | 120 (59) | | 73 (81) | | 47 (41) | | ≤0.001 | |  |
| Mental and behavioral disorders | 26 (13) | | 13 (14) | | 13 (11) | | 0.50 | |  |
| Cancer | 15 (7) | | 4 (4) | | 11 (10) | | 0.16 | |  |
| Congenital malformations, deformations and  chromosomal abnormalities | 15 (7) | | 9 (10) | | 6 (5) | | 0.19 | |  |
| Endocrine, nutritional and metabolic diseases | 11 (5) | | 8 (9) | | 3 (3) | | 0.048 | |  |
| Injury and other conditions with external causes | 3 (2) | | 3 (3) | | 0 | | - | |  |
| Other ^3^ | 6 (3) | | 2 (2) | | 4 (4) | | 0.60 | |  |
| Missing |  | 34 (17) | | 0 | | 34 (30) | | - | |

Abbreviations: CBD, cannabidiol; THC, tetrahydrocannabinol

^1^ p-value calculated by excluding the missing

^2^ p-values for each diagnosis by ICD 10 vs. no disease (reference), were calculated using chi-squared of Fisher

^3^ Other: 2 diseases of the blood, 1 disease of the digestive system, 1 infectious and parasitic disease, 1 disease of the musculoskeletal system and connective tissue, 1 disease of the skin and subcutaneous tissue

**Table S3:** Characteristics of participants with epilepsy only and with epilepsy and with additional diseases, who were treated with medical cannabis.

|  | **All**  n=66 (100%) | **Epilepsy with additional disease**  n=42 (100%) | **Epilepsy only**  n=24 (100%) | **p-value** |
| --- | --- | --- | --- | --- |
| **Sex** |  |  |  | 0.67 |
| Male | 38 (58) | 25 (60) | 13 (54) |  |
| Female | 28 (42) | 17 (40) | 11 (46) |  |
| **Median age at the first prescription in years (IQR)** | 10 (6-13) | 10.5 (7-15) | 8.5 (6-13) | 0.44 |
| **Type of epilepsy** |  |  |  |  |
| *Treatment resistant epilepsy* | 20 (30) | 14 (33) | 6 (24) |  |
| *Dravet-Syndrome* | 7 (11) | 0 | 7 (29) |  |
| *Lennox-Gastaut-Syndrome (LGS)* | 7 (11) | 3 (7) | 4 (17) |  |
| **Preparation ^1^** |  |  |  | 0.001 |
| THC | 16 (24) | 14 (33) | 2 (8) |  |
| CBD only | 50 (76) | 28 (67) | 22 (92) |  |
| **Frequency of seizures ^2^** |  |  |  | 0.73 |
| Much less | 13 (20) | 9 (21) | 4 (17) |  |
| Less | 23 (35) | 14 (33) | 9 (38) |  |
| Unchanged | 26 (39) | 15 (36) | 11 (46) |  |
| More | 1 (2) | 1 (2) | 0 |  |
| Much more | 3 (5) | 3 (7) | 0 |  |
| **Exact monthly costs in USD, median (IQR)** | 663 (250-2,000) | 750 (200-2,487) | 613 (350-1,400) | 0.89 |
| Number of observations | 26 | 16 | 10 |  |

Abbreviations: CBD, cannabidiol; THC, tetrahydrocannabinol

^1^ Six patients who received both THC and pure CBD were assigned to THC

^2^ as judged by the caregivers before and after treatment

p values are derived from chi-square or Fisher's exact tests as appropriat

**Table S4:** Characteristics of the 90 included participants by “CBD only”, “THC all”, “THC only” and “THC and CBD”.

|  |  |  |  | **THC** | | |
| --- | --- | --- | --- | --- | --- | --- |
|  | **Total** |  | **CBD only** | **THC all^1^** | **THC only^2^** | **THC and CBD^2^** |
| **Total** | n=90 (100%) |  | n=51 (100%) | n=39 (100%) | n=17 (100%) | n=22 (100%) |
| **Sex** |  |  |  |  |  |  |
| Male | 58 (64) |  | 34 (67) | 24 (62) | 12 (71) | 12 (55) |
| Female | 32 (36) |  | 17 (33) | 15 (39) | 5 (29) | 10 (45) |
| **Median age at the first prescription in years (IQR)** | 11.5 (6-15) |  | 9 (6-14) | 14 (9-16) | 14 (9-16) | 13.5 (9-16) |
| **Number of diagnoses** |  |  |  |  |  |  |
| One | 39 (43) |  | 26 (51) | 13 (33) |  |  |
| Two or more | 51 (57) |  | 25 (49) | 26 (67) |  |  |
| **Diagnosis** | |  |  |  |  |  |
| Epilepsy | 66 (73) |  | 44 (86) | 22 (56) | 8 (47) | 14 (64) |
| *Drug resistant epilepsy* | *20 (22)* |  | *17 (33)* | *3 (8)* |  |  |
| *Dravet-Syndrome* | *7 8)* |  | *6 (12)* | *1 (3)* |  |  |
| *Lennox-Gastaut-Syndrome (LGS)* | *7 (8)* |  | *7 (14)* | *-* |  |  |
| *Absences* | *4 (4)* |  | *1 (2)* | *3 (8)* |  |  |
| Cerebral palsy | 32 (36) |  | 13 (26) | 19 (49) | 9 (56) | 10 (45) |
| Encephalopathy | 15 (17) |  | 9 (18) | 6 (15) | 5 (30) | 1 (5) |
| Metabolic disease | 8 (9) |  | 2 (4) | 6 (15) | 1 (0) | 5 (23) |
| Autism | 7 (8) |  | 6 (12) | 1 (3) | 0 | 1 (5) |
| Genetic disorder | 6 (7) |  | 2 (4) | 4 (10) | 0 | 4 (18) |
| Cancer | 4 (4) |  | 0 | 4 (10) | 2 (12) | 2 (9) |
| Tourette's syndrome | 3 (3) |  | 1 (2) | 2 (5) | 1 (6) | 1 (5) |
| Severe head injury | 3 (3) |  | 0 | 3 (8) | 1 (6) | 2 (9) |
| Other ^3^ | 8 (9) |  | 4 (8) | 4 (10) |  |  |
| **Symptoms/indication** |  |  |  |  |  |  |
| Seizure/Epilepsy | 60 (67) |  | 40 (78) | 20 (51) | 7 (41) | 13 (59) |
| Spasticity | 27 (30) |  | 7 (14) | 20 (51) | 9 (53) | 11 (50) |
| Pain | 26 (29) |  | 6 (12) | 20 (51) | 10 (59) | 10 (45) |
| Sleep disorder | 15 (17) |  | 10 (20) | 5 (13) | 2 (12) | 3 (14) |
| Lack of weight gain | 11 (12) |  | 3 (6) | 8 (21) | 5 (29) | 3 (14) |
| Anxiety disorders/behaviour | 10 (11) |  | 3 (6) | 7 (18) | 3 (18) | 4 (18) |
| Vomiting | 9 (10) |  | 2 (4) | 7 (18) | 3 (18) | 4 (18) |
| Nausea | 8 (9) |  | 1 (2) | 7 (18) | 3 (18) | 4 (18) |
| Loss of appetite | 7 (8) |  | 1 (2) | 6 (15) | 2 (12) | 4 (18) |
| ADHD, behaviour change | 5 (6) |  | 4 (8) | 1 (3) | 0 | 1 (5) |
| Inflammatory condition | 4 (4) |  | 2 (4) | 2 (5) | 1 (6) | 1 (5) |
| Tics | 3 (3) |  | 1 (2) | 2 (5) | 1 (6) | 1 (5) |
| Others | 3 (3) |  | - | 3 (8) | 1 (6) | 2 (9) |
| **Type of medical cannabis preparation, initial** |  |  |  |  |  |  |
| THC based preparation | 39 (43) |  |  | 39 (100) |  |  |
| *Dronabinol solution 2.5%.* | *20 (22)* |  | *-* | *20 (51)* | 17 (100) | 3 (14) |
| *Standardized cannabis tincture* | *10 (11)* |  | *-* | *10 (26)* |  | 10 (45) |
| *Standardized cannabis oil* | *9 (10)* |  | *-* | *9 (23)* |  | 9 (41) |
| CBD based preparation | 51 (57) |  | 51 (100) |  |  |  |
| *CBD 2.5%* | *20 (22)* |  | *20 (39)* | *-* |  |  |
| *CBD 5%* | *17 (19)* |  | *17 (33)* | *-* |  |  |
| *CBD 10%* | *14 (16)* |  | *14 (28)* | *-* |  |  |
| **Other co-medications** |  |  |  |  |  |  |
| Antiepileptic drugs | 60 (67) |  | 39 (77) | 21 (5) |  |  |
| Muscle relaxants | 10 (11) |  | 2 4) | 8 (21) |  |  |
| Analgesics and opiates | 10 (11) |  | 1 (2) | 9 (23) |  |  |
| Other drugs | 25 (28) |  | 11 (22) | 15 (39) |  |  |
| **Additional therapy** |  |  |  |  |  |  |
| Physical therapy | 63 (70) |  | 33 (65) | 30 (77) | 16 (94) | 14 (64) |
| Occupational therapy | 46 (51) |  | 29 (57) | 17 (44) | 10 (59) | 7 (32) |
| Osteopathy | 23 (26) |  | 13 (26) | 10 (26) | 7 (41) | 3 (14) |
| Speech therapy | 9 (10) |  | 5 (10) | 4 (10) | 3 (18) | 1 (5) |
| Traditional Chinese Medicine (TCM) | 6 (7) |  | 4 (8) | 2 (5) | 2 (12) | 0 |
| Homeopathy | 5 (6) |  | 3 (6) | 2 (5) | 0 | 2 (9) |
| Chiropractic | 5 (6) |  | 3 (6) | 2 (5) | 1 (6) | 1 (5) |
| Others ^3^ | 11 (12) |  | 6 (12) | 5 (13) | 3 (18) | 2 (9) |
| No answer | 13 (14) |  | 8 (16) | 5 (13) | 0 | 5 (23) |

Abbreviations: CBD, cannabidiol; THC, tetrahydrocannabinol; ADHD attention-deficit/hyperactivity disorder

^1^ Six patients who received both THC and pure CBD were assigned to THC

^2^ We found no statistical difference when comparing the two groups “THC only” and “THC and CBD” using fisher exact (p>0.06)

^3^ Others diseases include: 2 with neuropathic pain, 2 with AD(H)S, 2 with Epidermolysis sclerosis, 1 with Depression, 1 with Tuberous sclerosis

**Table 5:** Outcomes of medical cannabis therapy among 90 children and adolescents by “CBD only”, “THC all”, “THC only” and “THC and CBD”.

|  |  |  |  | **THC** | | |
| --- | --- | --- | --- | --- | --- | --- |
|  | **Total** |  | **CBD** | **THC all^1^** | **THC only**^2^ | **THC and CBD**^2^ |
|  | n=90 (100%) |  | n=51 (100%) | n=39, (100%) | n=17 (100%) | n= 22 (100%) |
| **Treatment success (caregiver perspective)** |  |  |  |  |  |  |
| Yes | 59 (66) |  | 33 (65) | 26 (67) | 11 (65) | 15 (68) |
| No | 28 (31) |  | 17 (33) | 11 (28) | 6 (35) | 5 (23) |
| Missing | 3 (3) |  | 1 (2) | 2 (5) | 0 | 2 (9) |
| **Treatment interruptions / treatment stop** |  |  |  |  |  |  |
| Yes | 39 (43) |  | 23 (45) | 16 (41) | 6 (35) | 10 (45) |
| No | 51 (57) |  | 28 (55) | 23 (59) | 11 (65) | 12 (55) |
| **Median time to first interruption / treatment stop in weeks (IQR)** | 8 (3-32) |  | 8 (3-20) | 6.5 (3.5-36) | 28 (3-53) | 6.5 (4-32) |
| *Number of observations* | *25* |  | *17* | *8* | 2 | 8 |
| **Reasons for interruption** |  |  |  |  |  |  |
| No improvement and stopped treatment | 22 (24) |  | 13 (26) | 9 (23) | 4 (24) | 5 (23) |
| Side effects | 18 (20) |  | 10 (20) | 8 (21) | 4 (24) | 4 (18) |
| Taking preparation via the tube | 17 (19) |  | 10 (20) | 7 (18) | 1 (6) | 6 (27) |
| Costs | 9 (10) |  | 9 (18) | 0 | 0 | 0 |
| Unpleasant smell/taste | 4 (4) |  | 2 (4) | 2 (5) | 0 | 2 (9) |
| **Side effects** | |  |  |  |  |  |
| No | 65 (72) |  | 37 (73) | 28 (72) | 11 (65) | 17 (77) |
| Yes | 25 (28) |  | 14 (27) | 11 (28) | 6 (35) | 5 (23) |
| Tiredness | 11 (12) |  | 5 (10) | 6 (15) | 3 (18) | 3 (14) |
| Sedation | 7 (8) |  | 3 (6) | 4 (10) | 3 (18) | 1 (5) |
| Dry mouth | 5 (6) |  | 3 (6) | 2 (5) | 1 (6) | 1 (5) |
| Nausea and vomiting | 4 (4) |  | 2 (4) | 2 (5) | 1 (6) | 1 (5) |
| Dizziness | 2 (2) |  | 0 | 2 (5) | 1 (6) | 1 (5) |
| Hallucinations | 2 (2) |  | 1 (2) | 1 (3) | 1 (6) | 0 |
| Impaired ability to think (cognitive changes) | 2 (2) |  | 0 | 2 (5) | 1 (6) | 1 (5) |
| Changed movement behaviour | 2 (2) |  | 1 (2) | 1 (3) | 1 (6) | 0 |
| Diarrheal | 2 (2) |  | 2 (4) | 0 | 0 | 0 |
| Red eyes | 1 (1) |  | 0 | 1 (3) | 0 | 1 (5) |
| Others | 6 (7) |  | 6 (12) | 0 | 0 | 0 |

Abbreviations: CBD, cannabidiol; THC, tetrahydrocannabinol

^1^ Six patients who received both THC and pure CBD were assigned to THC

^2^ We found no statistical difference when comparing the two groups “THC only” and “THC and CBD” using fisher exact (p>0.06)

**Table S6:** Observed treatment effects of medical cannabis use in children (from the caregivers' perspective).

| **Changes in:** | **Preparation** | **Much more** | **More** | **Less** | **Much less** | **No change** | **Not applicable** | **Preparation** | **Much more** | **More** | **Less** | **Much less** | **No change** | **Not applicable** |
| --- | --- | --- | --- | --- | --- | --- | --- | --- | --- | --- | --- | --- | --- | --- |
| *Frequency of seizures* | THC | 1 | 0 | 6 | 4 | 11 | 17 | CBD | 2 | 1 | 17 | 9 | 15 | 7 |
| *Sleepiness* | THC | 0 | 1 | 0 | 2 | 6 | 30 | CBD | 0 | 5 | 0 | 0 | 10 | 36 |
| *Weight change* | THC | 0 | 8 | 2 | 0 | 15 | 14 | CBD | 1 | 5 | 2 | 2 | 19 | 22 |
| *No of additional drugs* | THC | 0 | 0 | 8 | 4 | 16 | 11 | CBD | 2 | 2 | 4 | 3 | 20 | 20 |
| *Supportive therapy* | THC | 0 | 1 | 1 | 0 | 18 | 19 | CBD | 0 | 1 | 0 | 0 | 20 | 30 |
| *Pain* | THC | 0 | 0 | 15 | 4 | 9 | 10 | CBD | 0 | 0 | 3 | 2 | 14 | 32 |
| *Nausea and vomiting* | THC | 0 | 1 | 0 | 5 | 15 | 18 | CBD | 0 | 2 | 0 | 5 | 15 | 29 |
| *Fall asleep* | THC | 1 | 13 | 2 | 0 | 11 | 12 | CBD | 0 | 8 | 2 | 2 | 16 | 23 |
| *Sleep through* | THC | 2 | 12 | 1 | 0 | 12 | 12 | CBD | 1 | 11 | 2 | 2 | 12 | 13 |
| *Spasticity* | THC | 0 | 0 | 11 | 3 | 9 | 16 | CBD | 1 | 5 | 6 | 4 | 10 | 25 |
| *Satisfaction* | THC | 6 | 10 | 0 | 0 | 9 | 14 | CBD | 6 | 16 | 3 | 1 | 8 | 17 |
| *Aggressiveness* | THC | 1 | 1 | 0 | 3 | 11 | 23 | CBD | 2 | 5 | 3 | 4 | 10 | 27 |
| *Relaxation* | THC | 6 | 17 | 0 | 0 | 7 | 9 | CBD | 5 | 20 | 0 | 0 | 11 | 15 |
| *School performance* | THC | 0 | 4 | 0 | 1 | 9 | 25 | CBD | 1 | 7 | 0 | 0 | 16 | 27 |
| *Independence* | THC | 1 | 2 | 0 | 1 | 11 | 24 | CBD | 0 | 7 | 1 | 0 | 17 | 26 |
| *Joy of playing* | THC | 1 | 7 | 0 | 0 | 7 | 24 | CBD | 3 | 12 | 1 | 0 | 15 | 20 |
| *General condition* | THC | 5 | 17 | 1 | 0 | 6 | 10 | CBD | 8 | 23 | 0 | 0 | 8 | 12 |
| *Quality of life* | THC | 9 | 13 | 0 | 0 | 8 | 9 | CBD | 8 | 21 | 0 | 0 | 7 | 15 |

1. Complete paralysis of one or more parts of the body [↑](#footnote-ref-1)
2. ^2^ Family doctor: e.g. FMH General internal medicine, FMH Paediatrics [↑](#footnote-ref-2)
3. ^3^ Specialist: e.g. Neuropaediatrician, Anaesthesiologist [↑](#footnote-ref-3)
4. ^4^ Please circle the appropriate. [↑](#footnote-ref-4)
